# Have We Been Underestimating Modifiable Dementia Risk? An Alternative Approach for Calculating the Combined Population Attributable Fraction for Modifiable Dementia Risk Factors

**DOI:** 10.1093/aje/kwad138

**Published:** 2023-06-15

**Authors:** Heidi J Welberry, Christopher C Tisdell, Md. Hamidul Huque, Louisa R Jorm

**Keywords:** dementia, modifiable risk, population attributable fraction, risk factors

## Abstract

Estimating the fraction of dementia cases in a population attributable to a risk factor or combination of risk factors (the population attributable fraction (PAF)) informs the design and choice of dementia risk-reduction activities. It is directly relevant to dementia prevention policy and practice. Current methods employed widely in the dementia literature to combine PAFs for multiple dementia risk factors assume a multiplicative relationship between factors and rely on subjective criteria to develop weightings for risk factors. In this paper we present an alternative approach to calculating the PAF based on sums of individual risk. It incorporates individual risk factor interrelationships and enables a range of assumptions about the way in which multiple risk factors will combine to affect dementia risk. Applying this method to global data demonstrates that the previous estimate of 40% is potentially too conservative an estimate of modifiable dementia risk and would necessitate subadditive interaction between risk factors. We calculate a plausible conservative estimate of 55.7% (95% confidence interval: 55.2, 56.1) based on additive risk factor interaction.

## Abbreviations

CI: confidence interval
PAF: population attributable fraction
PCA: principal components analysis
RR: relative risk

Population attributable fractions (PAFs) are used extensively in epidemiology to describe the proportional contribution of a known risk factor to a disease. PAFs are used to estimate the proportion of cases that could be avoided if that risk factor were removed from the population. In dementia contexts, there is considerable interest in using PAFs to quantify the impact of modifiable risk factors on dementia and build the case for risk reduction activities (1–4).

For a single binary risk factor, the most common method of calculating a PAF is Levin’s formula (5):

(1) \begin{equation*} \mathrm{PAF}=\frac{p\left(R-1\right)}{p\left(R-1\right)+1}, \end{equation*}

where *p* is the risk factor prevalence in a population and *R* is the relative risk (RR) of the outcome in individuals with that risk factor versus those without it. It is assumed that no other factors are confounding the observed relationship between a risk factor and disease.

Levin’s formula (5) is a robust approach to estimating the PAF (6) when considering a single risk factor. However, Livingston et al. (4), in a recent comprehensive review, identified 12 potentially modifiable risk factors with strong evidence of a link with dementia and estimated that the combined PAF for these risk factors was 40%.

A challenge when quantifying the impact of an array of risk factors on a disease is that single–risk-factor PAFs cannot be added together to provide a combined PAF, as risk factors overlap (6). A robust approach is to jointly model the combined attributable risk related to multiple risk factors directly in large longitudinal cohort studies using regression techniques (7). This enables inclusion of interaction terms or stratification by risk factor subgroups to obtain the most accurate RRs for describing the combined effects of risk factors. However, while this approach has been applied in areas like cancer (8), in the case of dementia the lengthy amount of time between exposures and dementia diagnosis means that finding studies with adequate sample sizes and inclusion of all relevant modifiable risk factors across the life course is challenging (9).

Additionally, to accurately calculate PAFs, risk factor prevalence estimates ideally need to be population-based. For dementia this poses a challenge, as there is a long lag time between exposures and outcome, so population-based surveys do not generally capture both well, and dementia is difficult to measure due to the frailty of older age groups (9). To produce a reliable estimate of the modifiable dementia risk in the population, there remains a need to combine and apply RR estimates from longitudinal studies with population risk factor prevalence data.

Barnes and Yaffe (1) produced one of the first estimates of combined modifiable dementia risk using an extension of Levin’s formula based on an assumption that the risk factors are causally independent in the population and their combined effect multiplicative. Their formula is

(2) \begin{equation*} {\mathrm{PAF}}_{\mathrm{combined}}=1-\prod \limits_{j=1}^k\left(1-\mathrm{PAF}\left({x}_j\right)\right), \end{equation*}

where, using Levin’s formula, $\mathrm{PAF}({x}_j)$ is calculated for *k* risk factors and ${x}_j$ denotes the *j*th risk factor. This formula first identifies the proportion of cases not attributable to each risk factor $(1-\mathrm{PAF}({x}_j))$; it then finds the proportion of cases not attributable to any risk factor $\prod \limits_{j=1}^k(1-\mathrm{PAF}({x}_j))$, and then estimates the proportion remaining as the attributable fraction.

However, the assumption of independence of risk factors for dementia is almost certainly incorrect (1, 2). Dementia risk factors are known to be correlated—for example, hypertension and obesity. This prompted concern that this approach may overestimate attributable cases (2). To address this, Norton et al. (2) proposed weighting the individual PAFs before combining them. Mathematically this was summarized as

(3) \begin{equation*} {\mathrm{PAF}}_{\mathrm{combined}}=1-\prod \limits_{j=1}^k\left(1-\left[{w}_j\times \mathrm{PAF}\left({x}_j\right)\right]\right), \end{equation*}

where ${w}_j$ is a weight applied to the individual PAF for risk factor *j*:

\begin{align*} {w}_j=1-{\mathrm{communality}}_j. \end{align*}

The calculation of the communality for each risk factor (*j*) was undertaken via “principal components analysis of the inter-risk-factor tetrachoric correlation matrix. Specifically, it was calculated as the square of the loadings on the first two principal components since both had eigenvalues greater than one—the Kaiser criterion for selecting the number of components to extract” (2, p. 790). That is, the individual PAFs are weighted to reduce the contribution of risk factors that have a lot of overlap with other risk factors.

Norton et al.’s approach has had widespread appeal, being used to produce the widely cited estimate of 40% calculated by Livingston et al. (4) and to provide an adjusted combined PAF in many other dementia studies (10–17). The proposed benefit of weighting is to provide a more realistic estimate than methods that do not account for the nonindependence of risk factors (2).

However, this assertion and methodology should be further scrutinized. When considering the unweighted formula used by Barnes and Yaffe (1), there are 2 assumptions being made. First, the risk factors are occurring independently in the population—which is not likely to be the case for dementia. Second, the combined effect of the risk factors is multiplicative. Multiplicative interactions are plausible ways in which risk factors may operate (18). For example, in the case of a multiplicative effect, having risk factor A with an RR of 1.5 and risk factor B with an RR of 2.0 will mean that the combined risk of having both risk factors A and B is a tripling of risk ($1.5\times 2.0=3.0$). Here, the absolute increase attributable to B will be greater among persons who have A ($1.5\to 3$; difference = 1.5) than among those who do not have A ($1.0\to 2.0$; difference = 1.0). Positive clustering of risk factors is therefore *likely to increase, not decrease,* the overall dementia risk in the population and may lead to underestimation of PAF if it is assumed that the risk factors are occurring independently.

Another practical issue is the difficulty in extracting consistent weights using principal components analysis (PCA). PCA operates by extracting linear combinations of the original risk factors to summarize the underlying variance in the data more efficiently (19). However, PCA is a data-driven technique that can be influenced by methodological choices, including the number of components retained. The Kaiser criterion of keeping only principal components with an eigenvalue greater than 1 is arbitrary and not necessarily the most reliable method of component selection (20). In the context of PAF calculation, PCA was used to derive weightings that summarize the proportion of variance a risk factor shares with the retained components from the PCA (2). The retention of more components necessarily leads to a larger proportion of shared variance (19), and so the communalities become larger, weightings smaller, and the resultant reduction in the combined PAF greater. Reliance on an arbitrary cutpoint given its material effect on the results is concerning, and when the exact combinations of risk factors are available, this PCA approach ignores valuable data.

We propose a new approach to calculating PAFs when individual-record–level data are available for risk factors but not for the outcome. Our approach does not rely on weightings and is mathematically equivalent to Levin’s formula (5) in the single–risk-factor case. It is also demonstrably equivalent to the method used by Barnes and Yaffe (1) under their same assumptions. Moreover, it enables a flexible method utilizing the population interrelationships of risk factors. It explicitly defines the assumption regarding the combined effect of the interaction between risk factors on the outcome disease so that multiple scenarios can be modeled. We demonstrate 2 of these—a multiplicative effect and an additive effect—using worked examples.

## PROPOSED APPROACH

We demonstrate that the PAF can be viewed as the quotient of 2 sums: individual risk attributable to the risk factor(s) and total risk for that individual. We demonstrate this mathematically for the single–risk-factor case and then extend this to combine multiple risk factors, based on different assumptions regarding risk factor interaction.

### Individual-record approach to calculating a single–risk-factor PAF

Let ${x}_i$ be an indicator for a risk factor $x$ for the $i$th individual, with ${x}_i=1$ when the person has the risk factor and ${x}_i=0$ when they do not.

Let ${R}_x$ be the RR of disease in the presence of risk factor $x$, where the risk of disease in the absence of $x$ is 1.We assume that ${R}_x$ is a constant for all individuals.

 For a population of *n* people, we define the population prevalence (*p*) for risk factor *x* as

(4) \begin{equation*} p(x)=\frac{1}{n}\sum \limits_{i=1}^n{x}_i. \end{equation*}

We incorporate this into Levin’s formula (5) to produce the PAF for *x* in the disease of interest within our population:

$$ \mathrm{PAF}=\frac{\frac{1}{n}{\sum}_{i=1}^n{x}_i\left({R}_x-1\right)}{\left[\frac{1}{n}{\sum}_{i=1}^n{x}_i\left({R}_x-1\right)\right]+1}. $$

Using simple summation rules and observing that ${R}_x$ is a constant, we frame it at an individual level, namely

(5) \begin{align*}\notag \mathrm{PAF}&=\frac{\frac{1}{n}{\sum}_{i=1}^n{x}_i\left({R}_x-1\right)}{\frac{1}{n}\left(\left[{\sum}_{i=1}^n{x}_i\left({R}_x-1\right)\right]+n\right)}\\& \notag=\frac{\sum_{i=1}^n{x}_i\left({R}_x-1\right)}{\left[{\sum}_{i=1}^n{x}_i\left({R}_x-1\right)\right]+n}\\& =\frac{\sum_{i=1}^n{x}_i\left({R}_x-1\right)}{\sum_{i=1}^n\left[{x}_i\left({R}_x-1\right)+1\right]}. \end{align*}

Equation 5 has 2 components: The numerator $\sum \limits_{i=1}^n{x}_i\left(\!{R}_x\!-\!1\!\right)$ represents the total excess risk in the population due to exposure to the risk factor (let us call this $A$). ${A}_i$ is then the excess risk the *i*th person contributes due to their individual exposure. The denominator is the total risk in the population (let us call this $T$). ${T}_i$ is then defined as the total risk for the *i*th person. ${T}_i$ is relative to a baseline risk of 1 for an unexposed person. Importantly, both *A* and *T* comprise a sum involving individual risk.

We can then rewrite equation 5 as

(6) \begin{equation*} \mathrm{PAF}=\frac{\sum_{i=1}^n{A}_i}{\sum_{i=1}^n{T}_i}\ . \end{equation*}

When considering person *i*, the individual risk components are written as

$$ {T}_i={x}_i\left({R}_x-1\right)+1; $$

$$ {A}_i={x}_i\left({R}_x-1\right); $$

$$ {A}_i={T}_i-1. $$

### PAF with 2 binary risk factors

We now consider the case in which there are 2 binary risk factors $x$ and $y$. The *i*th person could have one of 4 combinations: *x_i_* = 0 and *y_i_* = 0; *x_i_* = 1 and *y_i_* = 0; *x_i_* = 0 and *y_i_* = 1; or *x_i_* = 1 and *y_i_* = 1. In the first scenario, there is no exposure and no excess risk, so ${T}_i=1;{A}_i=0.$ For the remaining scenarios, we could use RR estimates for all 3 subgroups if they are available; if not, we can use estimates for ${R}_x$ and ${R}_y$ and assume how the 2 risk factors will interact to produce ${R}_{xy}$.

First, we assume a multiplicative effect of having multiple risk factors, so that when both risk factors are present, ${R}_{xy}={R}_x\times {R}_y$ (21). This also represents the total RR ${T}_i$ for a person $i$ when ${x}_i=1\kern0.37em \mathrm{and}\ {y}_i=1$. The attributable risk ${A}_i$ for this person would then be ${R}_{xy}-1$, which is the difference in total risk from the baseline risk in the absence of risk factors (set at 1). If the *i*th individual has only risk factor $x$, but not $y$, the total risk ${T}_i$ for the individual will then be ${R}_x$ and the attributable risk would be ${R}_x-1$. Similarly, if the *i*th individual has risk factor $y$ but not $x$, the total risk ${T}_i$ for the individual will then be ${R}_y$ and the attributable risk will be ${R}_y-1$. Mathematically, ${A}_i$ and ${T}_i$ can be written as

$$ {A}_i=\left({x}_i\left({R}_x-1\right)+1\right)\times \left({y}_i\left({R}_y-1\right)+1\right)-1; $$

\begin{equation*} {T}_i=\left({x}_i\left({R}_x-1\right)+1\right)\times \left({y}_i\left({R}_y-1\right)+1\right).\end{equation*}

Using the form of equation 6 and these new definitions of ${T}_i$ and ${A}_i$ which combine 2 risk factors and assume a multiplicative effect of risk factors, we establish a combined PAF:

(7) \begin{align*} &{\mathrm{PAF}}_{\mathrm{comb}\_\mathrm{multi}}\notag\\&=\frac{\sum_{i=1}^n\left\{\left[\left({x}_i{R}_x-{x}_i+1\right)\times \left({y}_i{R}_y-{y}_i+1\right)\ \right]-1\right\}}{\sum_{i=1}^n\left[\left({x}_i{R}_x-{x}_i+1\right)\times \left({y}_i{R}_y-{y}_i+1\right)\right]}. \end{align*}

Second, we assume an additive effect of having multiple risk factors. When both risk factors are present, ${R}_{xy}={R}_x+{R}_y-1$, as described by Mehta and Preston (21). This also represents the total RR ${T}_i$ for a person $i$ when ${x}_i=1\kern0.37em \mathrm{and}\ {y}_i=1$. Because ${A}_i={T}_i-1$, the attributable risk ${A}_i$ for this person would then be ${R}_x+{R}_y-2$. As with a multiplicative assumption, if the *i*th individual has only risk factor $x$ but not $y$, then the total risk ${T}_i$ for the individual will be ${R}_x$ and the attributable risk will be ${R}_x-1$. Similarly, if the *i*th individual has risk factor $y$ but not $x$, the total risk ${T}_i$ for the individual will then be ${R}_y$ and the attributable risk will be ${R}_y-1$. Mathematically, ${A}_i$ and ${T}_i$ can be written as

\begin{align*} {A}_i&={x}_i\left({R}_x-1\right)+{y}_i\left({R}_y-1\right).\\{T}_i&={x}_i\left({R}_x-1\right)+{y}_i\left({R}_y-1\right)+1. \end{align*}

Again, using the form of equation 6 and the new definitions of ${T}_i$ and ${A}_i$ which combine 2 risk factors and assume an additive effect of risk factors, we establish an alternative combined PAF:

(8) \begin{align*} {\mathrm{PAF}}_{\mathrm{comb}\_\mathrm{add}}=\frac{\sum_{i=1}^n\left[{x}_i\left({R}_x-1\right)+{y}_i\left({R}_y-1\right)\right]\ }{\sum_{i=1}^n\left[{x}_i\left({R}_x-1\right)+{y}_i\left({R}_y-1\right)+1\ \right]}. \end{align*}

In the 2–binary-risk-factor case, we can view both equation 7 and equation 8 in terms of a contingency table such that ${n}_1,{n}_2,{n}_3,{n}_4$ represent the population counts within subgroups based on the values of $X$ and $Y$ and where ${n}_1+{n}_2+{n}_3+{n}_4=n$, the total population (Table 1; also see Web Table 1, available at https://doi.org/10.1093/aje/kwad138).

**Table 1:** Frequencies Within Population Subgroups Defined by 2 Binary Variables *x* and *y*

**Value of *x***	**Value of *y***		**Total**
	***y* = 1**	***y* = 0**	
*x* = 1	${n}_1$	${n}_2$	${n}_1+{n}_2$
*x* = 0	${n}_3$	${n}_4$	${n}_3+{n}_4$
Total	${n}_1+{n}_3$	${n}_2+{n}_4$	$n$

We demonstrate in Web Appendix 1 that we can then reframe the PAF as

(7.1) \begin{align*}& {\mathrm{PAF}}_{\mathrm{comb}\_\mathrm{multi}}=\frac{\begin{array}{c}\big[{n}_1\left({R}_{xy}-{R}_x-{R}_y+1\right)+\left({n}_1+{n}_2\right)\\\left({R}_x-1\right)+\left({n}_1+{n}_3\right)\left({R}_y-1\right)\big]\end{array}}
{\begin{array}{c}\big[{n}_1\left({R}_{xy}-{R}_x-{R}_y+1\right)+\left({n}_1+{n}_2\right)\\
\left({R}_x-1\right)+\left({n}_1+{n}_3\right)\left({R}_y-1\right)+n\big]\ \end{array}} \end{align*}

and that in the additive case $ {R}_{xy}={R}_x+{R}_y-1,$ so:

(8.1) \begin{align*}& {\mathrm{PAF}}_{\mathrm{comb}\_\mathrm{add}}\notag\\&=\frac{\left[\left({n}_1+{n}_2\right)\left({R}_x-1\right)+\left({n}_1+{n}_3\right)\left({R}_y-1\right)\right]}{\left[\left({n}_1+{n}_2\right)\left({R}_x-1\right)+\left({n}_1+{n}_3\right)\left({R}_y-1\right)+n\right]\ }. \end{align*}

### PAF with more than 2 binary risk factors

We can generalize equations 7 and 8 to situations with more than 2 risk factors. Let ${x}_{ij}$ be an indicator variable for the *i*th person and the *j*th risk factor in a population of *n* people and a set of *k* risk factors. We again assume that this variable has a corresponding RR ${R}_j$, constant across the population. We redefine ${T}_i$ and ${A}_i$ in the case of a multiplicative or additive effect of risk factors to be

\begin{align*} {T}_{\mathrm{comb}\_\mathrm{multi}i}&=\left[{\prod}_{j=1}^k\left({x}_{ij}{R}_j-{x}_{ij}+1\right)\right];\\{A}_{\mathrm{comb}\_\mathrm{multi}i}&=\left[{\prod}_{j=1}^k\left({x}_{ij}{R}_j-{x}_{ij}+1\right)\right]-1, \end{align*}

and

\begin{align*} {T}_{\mathrm{comb}\_\mathrm{add}i}&=\left[{\sum}_{j=1}^k{x}_{ij}\left({R}_j-1\right)\right]+1;\\{A}_{\mathrm{comb}\_\mathrm{add}i}&=\left[{\sum}_{j=1}^k{x}_{ij}\left({R}_j-1\right)\right]. \end{align*}

Using the form of equation 6 and these new definitions of ${T}_i$ and ${A}_i$, we can establish general forms of the combined PAF:

(9) \begin{align*} {\mathrm{PAF}}_{\mathrm{comb}\_\mathrm{multi}}=\frac{\sum_{i=1}^n\left\{\left[{\prod}_{j=1}^k\left({x}_{ij}{R}_j-{x}_{ij}+1\right)\right]-1\right\}}{\sum_{i=1}^n\left\{{\prod}_{j=1}^k\left({x}_{ij}{R}_j-{x}_{ij}+1\right)\right\}}; \end{align*}

(10) \begin{align*} {\mathrm{PAF}}_{\mathrm{comb}\_\mathrm{add}}=\frac{\sum_{i=1}^n\left\{{\sum}_{j=1}^k{x}_{ij}\left({R}_j-1\right)\right\}\ }{\sum_{i=1}^n\left\{\left[{\sum}_{j=1}^k{x}_{ij}\left({R}_j-1\right)\right]+1\right\}\ }. \end{align*}

We demonstrate in Web Appendix 2 that by substituting in the definition of risk factor prevalence from equation 4, the combined additive PAF simplifies to

(11) \begin{align*} {\mathrm{PAF}}_{\mathrm{comb}\_\mathrm{add}}=\frac{\sum_{j=1}^k{p}_j\left({R}_j-1\right)}{\left[{\sum}_{j=1}^k{p}_j\left({R}_j-1\right)\right]+1}. \end{align*}

This generalization of the combined additive PAF is independent of *i*, demonstrating that in this special case, the combined PAF will be dependent only on the overall population prevalence of each risk factor and the RR of each risk factor with regard to the outcome.

### Confidence interval

To calculate the 95% confidence interval (CI) for the combined PAF, we propose a 2-step bootstrap procedure which 1) creates replicate samples with replacement and 2) for each contained observation then samples the RRs parametrically based on the known/published RR and confidence limits (assuming the natural log of the RR follows a symmetrical normal distribution). R software code (R Foundation for Statistical Computing, Vienna, Austria) is included in Web Appendix 3.

## APPLICATION OF OUR NEW APPROACH

A hypothetical example is provided below to illustrate the use of this new approach for calculating a combined PAF when there are 2 risk factors.

Imagine there is a population with 10 people: 2 who smoke and 8 who do not smoke. We know from the literature that the RR (${R}_x$) for the effect of smoking on dementia is 1.6 (4). Of these people, 5 report hearing loss and 5 do not. We know from the literature that the RR (${R}_y$) for the effect of hearing loss on dementia is 1.9 (4). Of these 10 people, 1 person both is a smoker and has hearing loss. This is the proportion who would both smoke and have hearing loss by chance (0.2 × 0.5 = 0.1) and suggests that the 2 risk factors are independent.

**Table 2:** Hypothetical Data Set Demonstrating Individual Attributable and Total Risks in the Presence of 2 Binary Variables *x* and *y* and Assuming Multiplicative Risk Accumulation^a^

**Individual** $(i)$	**Smoking** **Prevalence** $\left({x}_i\right)$	**Hearing Loss** **Prevalence** $\left({y}_i\right)$	**Effect of Smoking** **on Dementia Risk** $\left({R}_x\right)$	**Effect of Hearing Loss on Dementia Risk** $\left({R}_y\right)$	**Attributable Risk** $\big({A}_i={T}_i-1\big)$	**Total Risk** $\big({T}_i=\left[{x}_i\big({R}_x-1\big)+1\right]\times \left[{y}_i\big({R}_y-1\big)+1\right]\big)$
1	1	1	1.6	1.9	2.04	3.04
2	1	0	1.6	1.9	0.60	1.60
3	0	1	1.6	1.9	0.90	1.90
4	0	0	1.6	1.9	0.00	1.00
5	0	1	1.6	1.9	0.90	1.90
6	0	0	1.6	1.9	0.00	1.00
7	0	1	1.6	1.9	0.90	1.90
8	0	0	1.6	1.9	0.00	1.00
9	0	1	1.6	1.9	0.90	1.90
10	0	0	1.6	1.9	0.00	1.00

^a^ Population: smoking prevalence = 0.2; hearing loss prevalence = 0.5; effect of smoking on dementia risk = 1.6; effect of hearing loss on dementia risk = 1.9; attributable risk = 6.24; total risk = 16.24.

We assume that the combined impact of hearing loss and smoking is multiplicative. The combined impact of having both risk factors is therefore 1.6 ×1.9 = 3.04. For people with both risk factors, their total dementia risk is 3.04. Of this risk, 2.04 is attributable to the presence of the 2 risk factors, since this is the difference between their total risk and the baseline risk if they had neither risk factor (3.04 − 1.00 = 2.04). Table 2 shows the hypothetical data arising from this population. We can calculate the overall combined PAF using the sums of the components (*A* and *T*) of the individual PAFs: 6.24/16.24 = 0.384, or 38.4%.

We can compare this with existing population-level methods by calculating the PAF for each risk factor individually using Levin’s formula (5):

\begin{align*} {\mathrm{PAF}}_{\mathrm{smoking}}=\frac{0.2\times \left(1.6-1\right)}{0.2\times \left(1.6-1\right)+1}=\frac{0.12}{1.12}=0.107. \end{align*}

$$ {\mathrm{PAF}}_{\mathrm{hearing}\ \mathrm{loss}}=\frac{0.5\times \left(1.9-1\right)}{0.5\times \left(1.9-1\right)+1}=\frac{0.45}{1.45}=0.310. $$

Given that the example in Table 2 includes risk factors that are independently distributed in the population, we should get an equivalent result using Barnes and Yaffe’s (1) combined formula:

$$ {\mathrm{PAF}}_{\mathrm{combined}}=1-\prod \limits_{j=1}^k\left(1-\mathrm{PAF}\left({x}_j\right)\right), $$

where ${x}_j$ denotes the *j*th risk factor in an array of *k* risk factors.

$$ {\mathrm{PAF}}_{\mathrm{combined}}=1-\left[\left(1-0.107\right)\times \left(1-0.310\right)\right] $$

$$ =1-\left(0.893\times 0.69\right)$$

$$ =\mathrm{0.384.}$$

While the result is equivalent in this case, the individual-risk–level method can provide a flexible method for use when the risk factors are *not* independently distributed. Two further hypothetical examples are provided. Web Appendix 4 demonstrates a case where the risk factors are positively correlated and shows that the PAF increases in this situation (see Web Table 2). Web Appendix 5 demonstrates that when assuming an additive interaction between risk factors, the PAF is lower and is independent of the correlation between risk factors (see Web Tables 3 and 4).

A dynamic PAF calculator for 2 binary risk factors is also provided in Web Appendix 6 based on equations 7.1 and 8.1. This enables exploration of the PAF for a range of sample sizes, prevalence, and overlap between risk factors.

**Figure 1:**
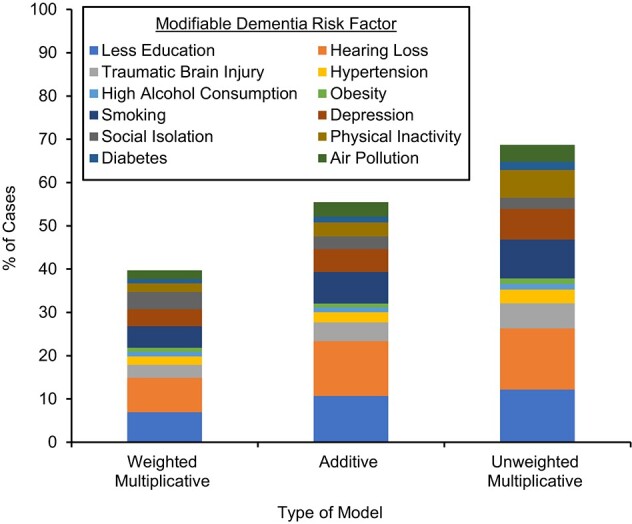
Comparison between multiplicative approaches and additive approaches to estimating the combined population attributable fraction (PAF) for multiple modifiable dementia risk factors. The weighted multiplicative model (reported by Livingston et al. (4)) and the unweighted multiplicative model (as per Barnes and Yaffe (1)) calculate a combined total PAF and then partition the total PAF into risk factor contributions based on the proportional split for each risk factor: individual PAF/sum of individual PAFs. The additive model (equation 11) calculates risk factor contributions directly by taking the attributable risk associated with a risk factor and dividing by the overall risk in the population: $\left[{p}_j\left({R}_j-1\right)\right]/\left(\left[\sum \limits_{j=1}^k{p}_j\left({R}_j-1\right)\right]+1\right)$. All risk factors are defined as per Livingston et al. (4).

Given that the combined additive PAF is reliant only on individual risk factor prevalence and RR estimates, we apply this approach described in equation 11 to the global data related to dementia prevention reported by Livingston et al. (4). Figure 1 and Web Appendix 7 contrast 3 different ways of combining the prevalence and risk estimates to produce PAFs. The adjusted combined PAF was reported as 40% by Livingston et al. (4) when following the weighted multiplicative method (equation 3) and as 69% when using Barnes and Yaffe’s (1) multiplicative formula (equation 2). This compares to 56% when the risk estimates are combined using an additive assumption as outlined in this paper (equation 11). Because there were no published confidence intervals for the prevalence estimates reported by Livingston et al. (4), we generated a synthetic data set using reported prevalence and a sample size of 5,000 (an approximate conservative sample size for a population health survey—for example, the National Health Survey in the United Kingdom). The mean combined additive PAF produced via this simulation was 55.7% (95% CI: 55.2, 56.1) (Web Appendix 3).

## DISCUSSION

In this paper we demonstrate, both mathematically and with examples, that the PAF for a single risk factor or a combination of risk factors can be calculated using the sums of individual risk in a population. For a single risk factor, this is equivalent to Levin’s formula (5). When individual-level data regarding risk factors exist—which is often the case in national population-based surveys—there are advantages to using this approach rather than calculating PAFs using population-level formulae.

For obtaining the PAF for multiple risk factors combined, we do not assume independence of the risk factors in the population, but instead account for the complexity and interrelatedness of risk factors in a robust way. This also facilitates meaningful comparisons of the combined PAF among subgroups with different risk factor clustering, or over time as risk factor prevalence and clustering change.

We incorporate a function which explicitly describes how we assume the multiple risk factors will operate in combination. This enables calculation of a range of combined PAFs based on evidence or assumptions about the mode of interaction between risk factors. Two scenarios were explored—scenarios where risk factors were combined on 1) a multiplicative basis and 2) an additive basis. Both are plausible ways in which modifiable dementia risks may interact. Mehta and Preston (21) observed that risks would be expected to operate additively when they pertain to different disease processes but may be superadditive or multiplicative in their effect when they operate on the same disease process. They found an additive effect of the relationship between smoking and obesity on mortality but a multiplicative effect of the interaction between sex and race. Di Angelantonio et al. (22) found a multiplicative effect of interaction between diabetes, stroke, and myocardial infarction (which all affect the cardiovascular system) on mortality. Very little research has explored modifiable risk factor *interaction* in relation to dementia, but Jang et al. (23) found that depression and cardiovascular disease interacted in a manner intermediate between additive and multiplicative in their combined effect on dementia.

Synergistic relationships are also possible (where the combination is supermultiplicative—greater than the product of the individual RRs). Ding et al. (24) noted in relation to mortality that certain combinations of modifiable risk factors, such as smoking and high alcohol consumption, may operate in this way. Conversely, risk factors may operate in a subadditive manner (where the addition of an extra risk factor makes no difference to risk, or only a small difference). Examples of subadditive interactions include gene-gene and gene-environment interactions, where the presence of a gene variant may be protective but its absence causative (and vice versa) (25). Paunio et al. (26) observed that risk of *Helicobacter pylori* infection increased with both age and alcohol consumption but that the interaction between these risk factors was subadditive.

The true way risk accumulates in the population is unlikely to be strictly multiplicative or additive. It may vary depending on the number and combination of risk factors. Continued accumulation of risk is likely to produce implausibly high approximations of the individual risk ratios for people with many risk factors. Incorporating additional parameters that constrain the RR within plausible limits may be useful extensions to the scenarios explored here. The explicit definition of the interaction assumption in the new approach we describe in this paper makes it flexible enough to accommodate any plausible scenario—an improvement on previous methods employed widely in the dementia literature.

This flexibility enables a demonstration that the adjusted combined PAF of 40% reported by Livingston et al. (4), and cited extensively, may underestimate modifiable dementia risk. For 40% to be a reasonable estimate, we would need to assume overall subadditive interaction between risk factors, suggesting that additional risk factors will increase risk only minimally. Is this plausible? While there is insufficient research to establish this categorically, a recent meta-analysis of studies that estimated risk for combinations of modifiable dementia risk factors showed that risk of dementia increased by 20% for the first risk factor (RR = 1.20, 95% CI: 1.04, 1.39), with an addition of 45% for the second risk factor (to RR = 1.65, 95% CI: 1.40, 1.94) and 55% (to RR = 2.21, 95% CI: 1.78, 2.73) for 3 or more risk factors (27). The effects at an individual study level ranged from risk factors combining in an additive way in the Uppsala Longitudinal Study of Adult Men (28) but in a multiplicative way in the Kaiser Permanente (29), Kungsholmen Project (30), and Cardiovascular Risk Factors, Aging and Dementia (CAIDE) Study (31) cohorts. These studies examined different combinations of modifiable risk factors, and it is unknown how risk may continue to accumulate with many risk factors. Nevertheless, these results are suggestive that the combined impact of dementia risk factors is at least additive. Therefore, our estimate of 56% based on an additive relationship between modifiable risk factors is likely to be a more realistic estimate of global modifiable dementia risk than the 40% previously reported (4)—although it may still potentially be conservative.

One of the main limitations with our new approach is that the method for combining risk assumes that an RR estimate represents causal risk related to a single risk factor in the absence of other risk factors. There are 2 different considerations that may interfere with this assumption—causal confounding and the nature of the interaction between risk factors (32). If the risk estimate for a risk factor is not confounded and if there is a multiplicative effect of combining risk factors (i.e., no effect modification), then individual RRs will provide reasonable estimates of the causal risk, as we are assuming that the relative effect of the risk factor is the same regardless of whether another risk factor is present.

For anything submultiplicative, such as an additive effect, this will not be the case. The *relative* effect of 1 risk factor is assumed to be lower among persons who have comorbid risk factors, since they are already at higher risk. In this situation, a single RR extracted from the literature is likely to underestimate the impact of the risk factor in the absence of other risk factors—if all other risk factors are adjusted for, it will provide the RR at the mean of those risk factors. The converse will be true if the combination of risk factors results in a supermultiplicative or synergistic effect. The implication of this is that if adjusted RRs are used and are assumed to combine additively, the overall combined PAF is likely to be conservative.

Levin’s formula (5) is known to be biased when using adjusted RRs in the presence of confounding (32), and this caveat also applies to our approach. Darrow and Steenland (32) observed that when a confounding factor is positively associated with a risk factor and outcome, an adjusted RR in Levin’s formula will introduce negative bias into the attributable fraction (it will be too conservative). The degree of bias introduced to the combined PAF for multiple risk factors is more complex to ascertain. For dementia, the risk factors under consideration are likely to cluster together, suggesting that the PAF will be conservative if fully adjusted RR estimates are used. The RRs reported by Livingston et al. (4) arose from meta-analyses of studies with different degrees of risk adjustment and different included covariates, measured in different ways. It is quite possible therefore that these estimates were not fully adjusted for all confounding factors and may have overstated the causal impact of these risk factors. This should be considered when evaluating *any* PAF reliant on these reported estimates.

Finally, it is worth considering the benefit of examining attributable risk at an individual level from a public health perspective. A combined PAF for multiple risk factors will provide the context for public health intervention, but knowing where in the population most of the risk lies is potentially very useful. The proposed approach does both. It combines risk estimates in an efficient way at a population level and simultaneously calculates the amount of attributable risk contributed by each person, informing the targeting of risk reduction activities as well as quantifying their potential impact.

In conclusion, this paper presents an alternative approach to calculating the PAF based on sums of individual risks. It directly incorporates risk factor interrelationships and provides a flexible framework for modeling the combined PAF. Applying this method to global data and assuming additive accumulation of risk provides a plausible conservative estimate for the combined modifiable PAF for dementia of 56%—16 percentage points higher than the previous 40% reported. This has widespread implications for modeling the potential health and economic impact of dementia risk reduction programs. This method is also broadly applicable to any area of research which aims to investigate the combined impact of exposures on an outcome.

## Supplementary Material

Web_Material_kwad138
