# Supplementary material for: Have We Been Underestimating Modifiable Dementia Risk? An Alternative Approach for Calculating the Combined Population Attributable Fraction for Modifiable Dementia Risk Factors
Source: Am J Epidemiol. 2023 Jun 15;192(10):1763–71. doi: 10.1093/aje/kwad138 (PMC10558200; doi:10.1093/aje/kwad138)
Supplement: Web_Material_kwad138 [file web_material_kwad138.zip › kwad138 Welberry Web Material corrected.docx]

WEB MATERIAL

Have We Been Underestimating Modifiable Dementia Risk? An Alternative Approach for Calculating the Combined Population Attributable Fraction for Modifiable Dementia Risk Factors

# Heidi J. Welberry, Christopher C. Tisdell, Md. Hamidul Huque, and Louisa R. Jorm

Contents

[**Web Appendix 1:** Reframing the two binary risk factor PAF in terms of a contingency table 2](#_Toc134112447)

**Web Table 1: Frequencies within population subgroups defined by two binary variables** $X$ **and** $Y$ **2**

[**Web Appendix 2:** Simplifying the combined additive PAF 3](#_Toc134112448)

[**Web Appendix 3:** R code for the PAF combining function to generate combined PAF estimates with 95% Confidence Intervals 4](#_Toc134112449)

[**Web Appendix 4:** Hypothetical scenario with two positively correlated risk factors and multiplicative impact of risk factors 9](#_Toc134112450)

**Web Table 2:** Hypothetical dataset demonstrating the individual attributable and total risk in the presence of two positively correlated binary variables x and y and assuming multiplicative risk accumulation 9

[**Web Appendix 5:** Hypothetical scenario with two binary risk factors and additive impact of risk factors 10](#_Toc134112451)

**Web Table 3:** Hypothetical dataset demonstrating the individual attributable and total risk in the presence of two positively correlated binary variables x and y and assuming additive risk accumulation 10

**Web Table 4:** Hypothetical dataset demonstrating the individual attributable and total risk in the presence of two negatively correlated binary variables x and y and assuming additive risk accumulation 11

[**Web Appendix 6:** PAF calculator for estimating the combined effect of two binary risk factors on an outcome [separate file] 12](#_Toc134112452)

[**Web Appendix 7:** Comparing estimates for the global modifiable PAF for dementia based on different assumptions regarding risk factor interaction 13](#_Toc134112453)

### **Web Appendix 1.** Reframing the two binary risk factor PAF in terms of a contingency table

$$PAF_{comb\_multi}=\frac{\sum_{i=1}^{n} {\{[(x}_{i}R_{x}-x_{i}+1) \times{(y}_{i}R_{y}-y_{i}+1)]-1\}}{\sum_{i=1}^{n} [{(x}_{i}R_{x}-x_{i}+1) \times{(y}_{i}R_{y}-y_{i}+1)]} .$$

$$=\frac{\sum_{i=1}^{n} {[x}_{i}y_{i}\left( R_{x}R_{y}-R_{x}-R_{y}+1 \right)+x_{i}\left( R_{x}-1 \right)+y_{i}(R_{y}-1)]}{\sum_{i=1}^{n} {[x}_{i}y_{i}\left( R_{x}R_{y}-R_{x}-R_{y}+1 \right)+x_{i}\left( R_{x}-1 \right)+y_{i}\left( R_{y}-1 \right)+1]}$$

We can then use the definition of prevalence from [4] to define $p\left( x \right)=\frac{1}{n}\sum_{i=1}^{n} x_{i}$; $p\left( y \right)=\frac{1}{n}\sum_{i=1}^{n} y_{i}$; $p\left( xy \right)=\frac{1}{n}\sum_{i=1}^{n} x_{i}y_{i}$ and show that:

$$PAF_{comb\_multi}=\frac{n\times\left[ p\left( xy \right)\left( R_{x}R_{y}-R_{x}-R_{y}+1 \right)+p\left( x \right)\left( R_{x}-1 \right)+p\left( y \right)\left( R_{y}-1 \right) \right]}{n\times\left[ p\left( xy \right)\left( R_{x}R_{y}-R_{x}-R_{y}+1 \right)+p\left( x \right)\left( R_{x}-1 \right)+p\left( y \right)\left( R_{y}-1 \right)+1 \right]}$$

$$=\frac{[p(xy)\left( R_{x}R_{y}-R_{x}-R_{y}+1 \right)+p(x)\left( R_{x}-1 \right)+p(y)(R_{y}-1)]}{[p\left( xy \right)\left( R_{x}R_{y}-R_{x}-R_{y}+1 \right)+p\left( x \right)\left( R_{x}-1 \right)+p\left( y \right)\left( R_{y}-1 \right)+1]}$$

In this case, with two binary risk factors, we can also view this expression in terms of a contingency table such that $n_{1},n_{2},n_{3},n_{4}$ represent the population counts within subgroups based on the values of *X* and *Y* and where $n_{1}+n_{2}+n_{3}+n_{4}=n$, the total population:

**Web Table 1. Frequencies within population subgroups defined by two binary variables** $\boldsymbol{X}$ **and** $\boldsymbol{Y}$

|  | **Y=1** | **Y=0** | **Total** |
| --- | --- | --- | --- |
| **X=1** | $n_{1}$ | $n_{2}$ | $n_{1}+n_{2}$ |
| **X=0** | $n_{3}$ | $n_{4}$ | $n_{3}+n_{4}$ |
| **Total** | $n_{1}+n_{3}$ | $n_{2}+n_{4}$ | $n$ |

We can then reframe the PAFs as:

[7.1]

$$PAF_{comb\_multi}=\frac{[n_{1}\left( R_{xy}-R_{x}-R_{y}+1 \right)+(n_{1}+n_{2})\left( R_{x}-1 \right)+(n_{1}+n_{3})(R_{y}-1)]}{[n_{1}\left( R_{xy}-R_{x}-R_{y}+1 \right)+(n_{1}+n_{2})\left( R_{x}-1 \right)+(n_{1}+n_{3})(R_{y}-1)+n]}$$

and:

[8.1]

$$PAF_{comb\_add}=\frac{[(n_{1}+n_{2})\left( R_{x}-1 \right)+(n_{1}+n_{3})(R_{y}-1)]}{[(n_{1}+n_{2})\left( R_{x}-1 \right)+(n_{1}+n_{3})(R_{y}-1)+n]} .$$

### **Web Appendix 2.** Simplifying the combined additive PAF

$$PAF_{comb\_add}=\frac{\sum_{i=1}^{n} \left\{ \sum_{j=1}^{k} x_{ij}\left( R_{j}-1 \right) \right\}}{\sum_{i=1}^{n} \left\{ \left[ \sum_{j=1}^{k} x_{ij}\left( R_{j}-1 \right) \right]+1 \right\}} .$$

Because this is a combination of sums, we can simplify this by rearranging:

$$PAF_{comb\_add}=\frac{\sum_{i=1}^{n} \left\{ \sum_{j=1}^{k} x_{ij}\left( R_{j}-1 \right) \right\}}{\sum_{i=1}^{n} \left[ \sum_{j=1}^{k} x_{ij}\left( R_{j}-1 \right) \right]+\sum_{i=1}^{n} 1}$$

$$=\frac{\sum_{j=1}^{k} \left\{ \sum_{i=1}^{n} x_{ij}\left( R_{j}-1 \right) \right\}}{\sum_{j=1}^{k} \left[ \sum_{i=1}^{n} x_{ij}\left( R_{j}-1 \right) \right]+n}$$

$$=\frac{\sum_{j=1}^{k} \left\{ \left( R_{j}-1 \right)\times\sum_{i=1}^{n} x_{ij} \right\}}{\sum_{j=1}^{k} \left[ \left( R_{j}-1 \right)\times\sum_{i=1}^{n} x_{ij} \right]+n}.$$

We can then use the definition of prevalence from [4] $p\left( x \right)=\frac{1}{n}\sum_{i=1}^{n} x_{i ,}$and define $p_{j}$ as the population prevalence for the $j$th risk factor. Applying this we get:

$$\frac{\sum_{j=1}^{k} \left\{ \left( R_{j}-1 \right)\times\sum_{i=1}^{n} x_{ij} \right\}}{\sum_{j=1}^{k} \left[ \left( R_{j}-1 \right)\times\sum_{i=1}^{n} x_{ij} \right]+n}=\frac{\sum_{j=1}^{k} \left\{ \left( R_{j}-1 \right)\times np_{j} \right\}}{\sum_{j=1}^{k} \left[ \left( R_{j}-1 \right)\times np_{j} \right]+n}.$$

This can then be simplified:

Equation [11]

$$\frac{\sum_{j=1}^{k} \left\{ \left( R_{j}-1 \right)\times np_{j} \right\}}{\sum_{j=1}^{k} \left[ \left( R_{j}-1 \right)\times np_{j} \right]+n}=\frac{n\sum_{j=1}^{k} \left\{ \left( R_{j}-1 \right)\times p_{j} \right\}}{n\sum_{j=1}^{k} \left[ \left( R_{j}-1 \right)\times p_{j} \right]+n}$$

$$=\frac{\sum_{j=1}^{k} p_{j}(R_{j}-1)}{\left[ \sum_{j=1}^{k} p_{j}(R_{j}-1) \right]+1}.$$

### **Web Appendix 3.** R code for the PAF combining function to generate combined PAF estimates with 95% confidence intervals

##############################################################################

## Title: PAF combining function - two step Bootstrap & Parametric ##

## sampling ##

## Purpose: This function combines Risk Ratios and sample Prevalence ##

## to provide a combined PAF for multiple risk factors ##

## with confidence intervals. ##

## It assumes causal independence of risk factors. ##

## It provides options to apply either a multiplicative or ##

## additive accumulation of risk ##

## Author: H Welberry ##

## Date: April 2023 ##

##############################################################################

require(boot)

################################################################################

## PAF.comb Master function: ##

## <data> The sample data, 1 column per risk factor and 1 row per ##

## observation with presence or absence of the rf denoted by a ##

## 1 or 0 respectively. ##

## <rr> A matrix of risk ratios and their confidence intervals. Must ##

## include four columns ordered: rf name, estimate, LCL, UCL, ##

## <type> Defines the combining function:'A'=Additive,'M'=Multiplicative ##

## <rep> The number of replications for the bootstrap function ##

################################################################################

PAF.comb <- function(data,rr, type='A', rep=1000){

#set-up and re-format rr data

set.seed(100)

colnames(rr) <- c("rf", 'rr', "rr.95CI_L", "rr.95CI_U")

rr$rr <- as.numeric(rr$rr)

rr$rr.95CI_L <- as.numeric(rr$rr.95CI_L)

rr$rr.95CI_U <- as.numeric(rr$rr.95CI_U)

#estimate sd for RR

#first take the log

rr$lnrr <-log(rr$rr)

rr$lnrr.95CI_L <-log(rr$rr.95CI_L)

rr$lnrr.95CI_U <-log(rr$rr.95CI_U)

rr$lnsd_rr <- (rr$lnrr.95CI_U-rr$lnrr.95CI_L)/(1.96*2)

#Sub-function 1 to sample risk ratios and calculate

PAF.c.fn <- function(data,rr, type){

#Simulate RR data parametrically

r <- as.data.frame(1:nrow(data),)

for (j in 1:nrow(rr)) {

#the log(RR) distribution follows an approx normal distribution

lnrr.x <- rnorm(n,mean=rr[j,'lnrr'], sd=rr[j,'lnsd_rr'])

#and then take the antilog

rr.x <- exp(lnrr.x)

r[,j] <- as.data.frame(rr.x)

}

#multiplicatively

A.m <- as.data.frame(1:nrow(data),)

for (j in 1:nrow(rr)) {

A.m[,j] <- (data[,j]*r[,j]-data[,j]+1)

}

#additively

A.a <- as.data.frame(1:nrow(data),)

for (j in 1:nrow(rr)) {

A.a[,j] <- (data[,j]*(r[,j]-1))

}

#(i) multiplicative accumulation of risk

T_multi <- apply(A.m,1,FUN = prod)

A_multi <- T_multi-1

PAF.Multi<- sum(A_multi)/sum(T_multi)

#(ii) additive accumulation of risk

A_add <- apply(A.a,1,FUN = sum)

T_add <- A_add+1

PAF.Add <- sum(A_add)/sum(T_add)

# Return based on type selection 'A' = additive; 'M'= multiplicative

ifelse(type=='A',

return(PAF.Add),

return(PAF.Multi))

}

#Sub-Function 2 to define sample loop and extract PAF

fPAF <- function(data,i){

PAF.est <- PAF.c.fn(data[i,],rr,type)

return(PAF.est)

}

#Finally Run Bootstrap re-sampling to extract mean PAF and CI

res <- boot(data, fPAF, R=rep)

return(list(res,

boot.ci(res, type='norm')))

}

##############################

## Example implementation ##

##############################

##############################################################################

# Define the risk estimates to be used (Example provided is Based on Livingston

##et al. 2020):

rr <- as.data.frame(rbind(

c("Educ",1.6, 1.26,2.01),

c("Hearing",1.9, 1.38,2.73),

c("TBI",1.8, 1.5,2.2),

c("Hypertension", 1.6, 1.16,2.24),

c("Alcohol",1.2, 1.1,1.3),

c("Obesity", 1.6, 1.34,1.92),

c("Smoking", 1.6, 1.15,2.20),

c("Depression", 1.9, 1.55,2.33),

c("Isolation", 1.6, 1.32,1.85),

c("LowPA", 1.4, 1.2,1.7),

c("Diabetes", 1.5, 1.33,1.79),

c("AirPollution", 1.1, 1.1,1.1)))

###########################################################################

# Generate a synthetic dataset but with pre-defined prevalence of each rf ##

# based on the rates reported in: Livingston et al. 2020) ##

# NOTE: in practice this should be replaced with survey data representing ##

# binary indicator variables for presence/absence of each risk factor for ##

# each individual ##

###########################################################################

#define the number of observations

n <- 5000

#define the prevalence of risk factors (from Livingston et al.)

pr <- c(0.40, 0.32, 0.12, 0.09, 0.12, 0.03, 0.27, 0.132, 0.11, 0.18,

0.06, 0.75)

#generate data set

data <- as.data.frame(cbind(

"Educ"= c( rbinom(n, 1, pr[1])),

"Hearing"=c( rbinom(n, 1, pr[2])),

"TBI"= c(rbinom(n, 1, pr[3])),

"Hypertension"=c( rbinom(n, 1, pr[4])),

"Alcohol"=c(rbinom(n, 1, pr[5])),

"Obesity"= c(rbinom(n, 1, pr[6])),

"Smoking"=c(rbinom(n, 1, pr[7])),

"Depression"=c( rbinom(n, 1, pr[8])),

"Isolation"= c(rbinom(n, 1, pr[9])),

"LowPA"=c(rbinom(n, 1, pr[10])),

"Diabetes"=c( rbinom(n, 1, pr[11])),

"AirPollution"=c( rbinom(n, 1, pr[12]))))

#################################

## Apply the PAF.comb function ##

#################################

PAF.comb(data,rr, type='M', rep=1000 )

PAF.comb(data,rr, type='A', rep=1000 )

###########

## END ##

###########

##############################################################################

## References: ##

## Livingston G, Huntley J, Sommerlad A, et al. Dementia prevention, ##

## intervention, and care: 2020 report of the Lancet Commission. The Lancet.##

## 2020;396(10248):413-446. doi:10.1016/S0140-6736(20)30367-6 ##

## ##

##############################################################################

### **Web Appendix 4.** Hypothetical scenario with two positively correlated risk factors and multiplicative impact of risk factors

Another population has 10 people with similar prevalence of risk factors to the population in Table 2:

- 2 who smoke and 8 who do not smoke. The relative risk (*R_x_*) for smoking on dementia is 1.6.
- 5 who report hearing loss and 5 who do not. The relative risk (*R_y_*) for hearing loss on dementia is 1.9.

In this population though, 2 people both smoke and have hearing loss. This is HIGHER than the proportion who would both smoke and have hearing loss by chance ($0.2>0.2 \times0.5$) and suggests the two risk factors are **positively correlated**.

We again assume that the combined impact of hearing loss and smoking is multiplicative. The combined impact of having both risk factors is therefore $1.6 \times1.9 = 3.04$. **Web Table 2** demonstrates the hypothetical dataset arising from this population.

**Web Table 2. Hypothetical dataset demonstrating the individual attributable and total risk in the presence of two positively correlated binary variables** $\boldsymbol{x}$ **and** $\boldsymbol{y}$ **and assuming multiplicative risk accumulation**

| **Person number**  $\boldsymbol{(i)}$ | **Smoking**  **Prevalence**  **(**$\boldsymbol{x}_{\boldsymbol{i}}$ **)** | **Hearing loss**  **Prevalence**  **(**$\boldsymbol{y}_{\boldsymbol{i}}$ **)** | **Risk of smoking on Dementia**  **(**$\boldsymbol{R}_{\boldsymbol{x}}$**)** | **Risk of hearing loss on Dementia**  **(**$\boldsymbol{R}_{\boldsymbol{y}}$**)** | **Attributable Risk**  $\boldsymbol{A}_{\boldsymbol{i}}\boldsymbol{=}\boldsymbol{T}_{\boldsymbol{i}}\boldsymbol{-1}$ | **Total Risk**  $\boldsymbol{T}_{\boldsymbol{i}}\boldsymbol{=}\left( \boldsymbol{x}_{\boldsymbol{i}}\left( \boldsymbol{R}_{\boldsymbol{x}}\boldsymbol{-1} \right)\boldsymbol{+1} \right)\boldsymbol{\times}\left( \boldsymbol{y}_{\boldsymbol{i}}\left( \boldsymbol{R}_{\boldsymbol{y}}\boldsymbol{-1} \right)\boldsymbol{+1} \right)$ |
| --- | --- | --- | --- | --- | --- | --- |
| Population | (0.2) | (0.5) | (1.6) | (1.9) | (6.78) | (16.78) |
| 1 | 1 | 1 | 1.6 | 1.9 | 2.04 | 3.04 |
| 2 | 1 | 1 | 1.6 | 1.9 | 2.04 | 3.04 |
| 3 | 0 | 1 | 1.6 | 1.9 | 0.90 | 1.90 |
| 4 | 0 | 0 | 1.6 | 1.9 | 0.00 | 1.00 |
| 5 | 0 | 1 | 1.6 | 1.9 | 0.90 | 1.90 |
| 6 | 0 | 0 | 1.6 | 1.9 | 0.00 | 1.00 |
| 7 | 0 | 1 | 1.6 | 1.9 | 0.90 | 1.90 |
| 8 | 0 | 0 | 1.6 | 1.9 | 0.00 | 1.00 |
| 9 | 0 | 0 | 1.6 | 1.9 | 0.00 | 1.00 |
| 10 | 0 | 0 | 1.6 | 1.9 | 0.00 | 1.00 |

**The combined PAF is now higher in this population** at $6.78/16.78 = \boldsymbol{40.4\%}$**.**

When the individual components of risk are examined, we can see that when the RFs cluster together their impact becomes greater not lesser. It is important to note that this is only assuming that the effect of the risk factors on dementia is *multiplicative* and independent of their relationship with each other. That is, we are assuming that smoking raises risk of dementia by 60% among those without hearing loss *and* among those with hearing loss who are already at a higher risk.

It is quite possible that this multiplicative assumption is incorrect, and an alternate assumption is explored in Web Appendix 5.

### **Web Appendix 5.** Hypothetical scenario with two binary risk factors and additive impact of risk factors

Now consider the same 10 people as described in Web Appendix 4. Two people both smoke and have hearing loss suggesting the two risk factors are positively correlated.

However, we now assume that the combined impact of hearing loss and smoking is additive rather than multiplicative. That is smoking increases risk by 60% and then hearing loss increases it by an additional 90%. The combined impact of having both risk factors is therefore $1+0.6 + 0.9 = 2.5$.

**Web Table 3. Hypothetical dataset demonstrating the individual attributable and total risk in the presence of two positively correlated binary variables** $\boldsymbol{x}$ **and** $\boldsymbol{y}$ **and assuming additive risk accumulation**

| **Person Number**  $\boldsymbol{(i)}$ | **Smoking**  **Prevalence**  **(**$\boldsymbol{x}_{\boldsymbol{i}}$ **)** | **Hearing Loss Prevalence**  **(**$\boldsymbol{y}_{\boldsymbol{i}}$ **)** | **Risk of Smoking on Dementia**  **(**$\boldsymbol{R}_{\boldsymbol{x}}$**)** | **Risk of Hearing Loss on Dementia**  **(**$\boldsymbol{R}_{\boldsymbol{y}}$**)** | **Attributable Risk**  $\boldsymbol{A}_{\boldsymbol{i}}\boldsymbol{=}\boldsymbol{T}_{\boldsymbol{i}}\boldsymbol{-1}$ | **Total Risk**  $\boldsymbol{T}_{\boldsymbol{i}}\boldsymbol{=}\boldsymbol{x}_{\boldsymbol{i}}\boldsymbol{(}\boldsymbol{R}_{\boldsymbol{x}}\boldsymbol{-1)+}\boldsymbol{y}_{\boldsymbol{i}}\boldsymbol{(}\boldsymbol{R}_{\boldsymbol{y}}\boldsymbol{-1)+1}$ |
| --- | --- | --- | --- | --- | --- | --- |
| Population | (0.2) | (0.5) | (1.6) | (1.9) | (5.7) | (15.7) |
| 1 | 1 | 1 | 1.6 | 1.9 | 1.5 | 2.5 |
| 2 | 1 | 1 | 1.6 | 1.9 | 1.5 | 2.5 |
| 3 | 0 | 1 | 1.6 | 1.9 | 0.9 | 1.9 |
| 4 | 0 | 0 | 1.6 | 1.9 | 0.0 | 1.0 |
| 5 | 0 | 1 | 1.6 | 1.9 | 0.9 | 1.9 |
| 6 | 0 | 0 | 1.6 | 1.9 | 0.0 | 1.0 |
| 7 | 0 | 1 | 1.6 | 1.9 | 0.9 | 1.9 |
| 8 | 0 | 0 | 1.6 | 1.9 | 0.0 | 1.0 |
| 9 | 0 | 0 | 1.6 | 1.9 | 0.0 | 1.0 |
| 10 | 0 | 0 | 1.6 | 1.9 | 0.0 | 1.0 |

Based on this assumption, the combined PAF is now slightly lower at $5.7/15.7 = \boldsymbol{36.3\%}$**.**

Importantly, if we make an additive assumption, it does not matter whether the risk factors are independently distributed or not. The combined PAF will remain unchanged as we can see in the next table where there is different population of 10 people, with the same risk factor prevalence, but with zero people with both risk factors (i.e., a negative correlation between risk factors) (Web Table 4).

**Web Table 4. Hypothetical dataset demonstrating the individual attributable and total risk in the presence of two negatively correlated binary variables** $\boldsymbol{x}$ **and** $\boldsymbol{y}$ **and assuming additive risk accumulation**

| **Person Number**  $\boldsymbol{(i)}$ | **Smoking**  **Prevalence**  **(**$\boldsymbol{x}_{\boldsymbol{i}}$ **)** | **Hearing Loss Prevalence**  **(**$\boldsymbol{y}_{\boldsymbol{i}}$ **)** | **Risk of Smoking on Dementia**  **(**$\boldsymbol{R}_{\boldsymbol{x}}$**)** | **Risk of Hearing Loss on Dementia**  **(**$\boldsymbol{R}_{\boldsymbol{y}}$**)** | **Attributable Risk**  $\boldsymbol{A}_{\boldsymbol{i}}\boldsymbol{=}\boldsymbol{T}_{\boldsymbol{i}}\boldsymbol{-1}$ | **Total Risk**  $\boldsymbol{T}_{\boldsymbol{i}}\boldsymbol{=}\boldsymbol{x}_{\boldsymbol{i}}\boldsymbol{(}\boldsymbol{R}_{\boldsymbol{x}}\boldsymbol{-1)+}\boldsymbol{y}_{\boldsymbol{i}}\boldsymbol{(}\boldsymbol{R}_{\boldsymbol{y}}\boldsymbol{-1)+1}$ |
| --- | --- | --- | --- | --- | --- | --- |
| Population | (0.2) | (0.5) | (1.6) | (1.9) | (5.7) | (15.7) |
| 1 | 1 | 0 | 1.6 | 1.9 | 0.6 | 1.6 |
| 2 | 1 | 0 | 1.6 | 1.9 | 0.6 | 1.6 |
| 3 | 0 | 1 | 1.6 | 1.9 | 0.9 | 1.9 |
| 4 | 0 | 1 | 1.6 | 1.9 | 0.9 | 1.9 |
| 5 | 0 | 1 | 1.6 | 1.9 | 0.9 | 1.9 |
| 6 | 0 | 0 | 1.6 | 1.9 | 0.0 | 1.0 |
| 7 | 0 | 1 | 1.6 | 1.9 | 0.9 | 1.9 |
| 8 | 0 | 0 | 1.6 | 1.9 | 0.0 | 1.0 |
| 9 | 0 | 1 | 1.6 | 1.9 | 0.9 | 1.9 |
| 10 | 0 | 0 | 1.6 | 1.9 | 0.0 | 1.0 |

The combined PAF remains at $5.7/15.7 = \boldsymbol{36.3\%}$**.**

We can also demonstrate the equivalence with equation [11] that shows individual level data regarding risk factors is not required when assuming an additive relationship:

$$PAF_{comb\_add}=\frac{\sum_{j=1}^{k} p_{j}\left( R_{j}-1 \right)}{\left[ \sum_{j=1}^{k} p_{j}\left( R_{j}-1 \right) \right]+1}$$

$$=\frac{0.2\left( 1.6-1 \right)+0.5\left( 1.9-1 \right)}{0.2\left( 1.6-1 \right)+0.5\left( 1.9-1 \right)+1}$$

$$=\frac{0.57}{1.57}=0.363$$

### **Web Appendix 6.** PAF calculator for estimating the combined effect of two binary risk factors on an outcome

Web Appendix 6 is provided as a separate Excel file.

### **Web Appendix 7.** Comparing estimates for the global modifiable PAF for dementia based on different assumptions regarding risk factor interaction

1. The Unadjusted Multiplicative total uses the individual unadjusted PAFs reported within Livingston. et al. and combines them as per the Barnes and Yaffe method:

$$PAF_{\mathrm{combined}}=1-\prod_{j=1}^{k} \left( 1-PAF\left( x_{j} \right) \right)$$

$$=1-\left( 1-0.19 \right)\times\left( 1-0.22 \right)\times\left( 1-0.09 \right)\times\left( 1-0.05 \right)\times\left( 1-0.02 \right)\times\left( 1-0.02 \right)\times\left( 1-0.14 \right)\times\left( 1-0.11 \right)\times\left( 1-0.04 \right)\times\left( 1-0.10 \right)\times\left( 1-0.03 \right)\times\left( 1-0.06 \right)$$

$$=1-0.313$$

$$=0.687$$

1. The Additive total is based on:

$$PAF_{comb\_add}=\frac{\sum_{j=1}^{k} p_{j}(R_{j}-1)}{\left[ \sum_{j=1}^{k} p_{j}(R_{j}-1) \right]+1}$$

$$=\left[ 0.40\left( 1.6-1 \right)+0.32\left( 1.9-1 \right)+0.12\left( 1.8-1 \right)+0.09\left( 1.6-1 \right)+0.12\left( 1.2-1 \right)+0.03\left( 1.6-1 \right)+0.27\left( 1.6-1 \right)+0.13\left( 1.9-1 \right)+0.11\left( 1.6-1 \right)+0.18\left( 1.4-1 \right)+0.06\left( 1.5-1 \right)+0.75\left( 1.1-1 \right) \right]$$

$$/$$

$$\left[ 0.40\left( 1.6-1 \right)+0.32\left( 1.9-1 \right)+0.12\left( 1.8-1 \right)+0.09\left( 1.6-1 \right)+0.12\left( 1.2-1 \right)+0.03\left( 1.6-1 \right)+0.27\left( 1.6-1 \right)+0.13\left( 1.9-1 \right)+0.11\left( 1.6-1 \right)+0.18\left( 1.4-1 \right)+0.06\left( 1.5-1 \right)+0.75\left( 1.1-1 \right) \right]+1$$

$$=1.25/2.25$$

$$=0.555$$
